# Exploring social determinants of health in the context of metabolic and circadian influences on new hip fracture risk: longitudinal insights from CHARLS

**DOI:** 10.1186/s12889-025-25126-5

**Published:** 2025-11-21

**Authors:** Peng Hu, Hongliang Du

**Affiliations:** Department of Orthopaedic Surgery, Shanxi Bethune Hospital, Shanxi Academy of Medical Sciences, Third Hospital of Shanxi Medical University, Tongji Shanxi Hospital, No.99, Longcheng Street, Taiyuan, Shanxi Province 030032 China

**Keywords:** Social determinants of health, Metabolic syndrome, Circadian rhythm syndrome, Hip fractures, CHARLS

## Abstract

**Background:**

The risk of hip fractures is closely intertwined with social determinants of health (SDHs), metabolic factors, and circadian rhythm-related variables. Understanding the combined effects of these factors, particularly in the elderly population of China, has substantial public health implications.

**Methods:**

This study utilized data from the China Health and Retirement Longitudinal Study (CHARLS, 2011–2020), which included 11,928 individuals aged 45 years and older, all free from a history of hip fractures. Kaplan-Meier survival curves, multivariable Cox proportional hazards models, and restricted cubic spline (RCS) analysis were applied to explore the associations between SDHs and hip fracture risk, stratified by metabolic syndrome (MetS) and circadian rhythm syndrome (CircS). Sensitivity analyses and receiver operating characteristic (ROC) curves were conducted to validate the findings and assess the predictive performance.

**Results:**

Higher SDHs scores were consistently associated with a reduced risk of hip fractures, particularly among individuals with MetS and CircS. For each unit increase in SDHs, the risk of first hip fracture was reduced by 14% (HR = 0.86; 95%CI, 0.77–0.95; *P* < 0.003). RCS analysis revealed a significant relationship between SDHs and the incidence of hip fractures across various metabolic and circadian rhythm subgroups (all P-values < 0.05, non-linear *P* > 0.05). Kaplan-Meier analysis demonstrated statistically significant differences in hip fracture incidence between SDHs groups (log-rank *P* < 0.001). ROC curve analysis demonstrated superior predictive accuracy for SDHs, with an AUC of 0.723 and 0.731, particularly among individuals with MetS and CircS.

**Conclusions:**

SDHs are independently associated with a decreased risk of hip fractures, with a particularly pronounced effect observed in individuals with MetS and CircS. These findings underscore the critical importance of addressing both social and metabolic factors in the prevention of hip fractures, especially within aging populations.

**Supplementary Information:**

The online version contains supplementary material available at 10.1186/s12889-025-25126-5.

## Introduction

Hip fractures pose a significant public health challenge, particularly among the elderly, given their association with increased morbidity, mortality, and long-term disability. As the global population continues to age, understanding the multifactorial risk factors contributing to hip fractures has become increasingly essential. While osteoporosis and falls have long been identified as primary risk factors, a growing body of evidence underscores the equally critical role of social determinants of health (SDHs) in influencing the likelihood of such injuries [1–3]. These determinants encompass a broad array of factors, including socioeconomic status, education, social support, and neighborhood environment, all of which shape an individual’s health trajectory across the lifespan. Of particular concern are metabolic factors such as obesity, diabetes, and metabolic syndrome, compounded by disruptions in circadian rhythms, which are emerging as key contributors to bone health and fracture risk [4–6]. 

In recent years, the significance of metabolic health in the risk of hip fractures has garnered increasing attention. Metabolic conditions such as diabetes and obesity adversely affect overall health by influencing bone mineral density, muscle mass, and balance, all of which directly heighten the risk of falls and fractures [4, 7]. For example, insulin resistance and chronic inflammation associated with metabolic syndrome (MetS) can reduce bone formation and increase bone resorption, thereby enhancing fracture susceptibility. Moreover, these metabolic disturbances often coincide with additional risk factors, including malnutrition and physical inactivity, which are themselves shaped by SDHs such as income, education, and access to healthcare [8–10]. Therefore, examining the interplay between metabolic health and social factors offers a more comprehensive understanding of hip fracture risk, particularly in elderly populations. Equally important is the role of circadian rhythm (CircS) in skeletal health. Disruptions to the circadian system, whether due to irregular sleep patterns, shift work, or aging, have been associated with a range of health conditions, including osteoporosis and an increased risk of fractures [11, 12]. The circadian rhythm governs numerous biological processes, such as hormonal regulation, metabolic function, and tissue repair, all of which influence bone strength and fracture vulnerability [13, 14]. The relationship between circadian rhythms and bone health is particularly salient in older adults, as age-related changes in circadian function can exacerbate metabolic disturbances and increase skeletal fragility [15–17]. 

The longitudinal data from the China Health and Retirement Longitudinal Study (CHARLS) provide a unique and invaluable opportunity to explore the intricate and multifaceted interactions between SDHs, metabolic health, circadian rhythm, and hip fracture risk. With its comprehensive dataset, which includes a wide array of socioeconomic, demographic, and health factors, CHARLS facilitates a thorough examination of how these variables intersect to influence aging-related outcomes such as hip fractures. By integrating data on metabolic conditions, such as diabetes, obesity, and metabolic syndrome, alongside information on sleep patterns, circadian rhythm disruptions, and social inequities, this study offers a holistic perspective on the mechanisms underlying increased fracture risk.

## Methods

### Study design and population

**Fig. 1:**
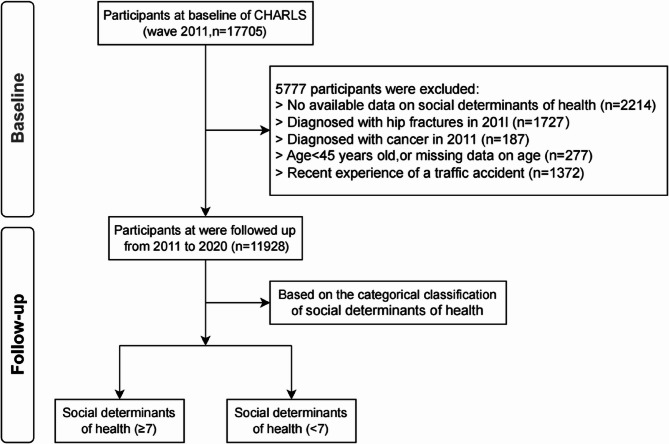
Flowchart of participants’ selection. Abbreviation: CHARLS, China health and retirement longitudinal study

This study utilized data from the CHARLS, a nationally representative survey of individuals aged 45 and above across 23 provinces in China [18]. The CHARLS survey employed a multi-stage random sampling method, stratified by proportional probability and size. Initially, 17,705 participants were enrolled in 2011. However, 5,777 participants were excluded for the following reasons: (1) missing data on SDHs (*n* = 2,214), (2) a history of hip fractures in 2011 (*n* = 1,727), (3) a diagnosis of cancer in 2011 (*n* = 187), (4) age under 45 years or missing age data (*n* = 277), and (5) a recent traffic accident (*n* = 1,372). The final cohort comprised 11,928 participants, who were followed from 2011 to 2020 (Fig. 1). All participants provided written informed consent, and the study was approved by the Biomedical Ethics Review Committee of Peking University (IRB00001052-11015). Detailed information on the CHARLS study design and data collection is available on the official website (http://charls.pku.edu.cn/en*).*

### Ascertainment of SDHs, MetS and circs

Drawing from the World Health Organization and Healthy People 2030 frameworks, the SDHs score emphasizes the multidimensional nature of social well-being [19, 20]. In addition, recent empirical research that illustrates the utility of composite SDHs indices in epidemiological studies influenced the design (Zhong et al., 2024) [21].The Chinese version of SDHs described in the CHARLS encompasses a wide array of socioeconomic and health-related factors that have a profound impact on individual well-being. These determinants include economic stability (such as housing, income, and employment), social support (covering marital status, social participation, and living arrangements), access to healthcare (encompassing insurance coverage and proximity to medical facilities), and personal health conditions (such as depression and educational attainment). Collectively, these factors exert a significant influence on the health and welfare of elderly individuals in China, providing essential insights into the broader socioeconomic context that shapes health outcomes. Housing status is divided into two categories: homeowners score 1, while renters score 0, underscoring the link between housing stability, economic well-being, and access to essential resources. Household income was dichotomized into two categories: individuals from households with annual per capita income below ¥9,000 (Supplementary materials) were assigned a value of 0, while those at or above this threshold were assigned a value of 1. This categorization was designed to reflect the role of economic disadvantage as a key constraint on access to health-related resources (https://www.stats.gov.cn/). Employment status follows a similar classification: employed or retired individuals score 1, while unemployed individuals score 0, reflecting the considerable influence of employment on access to health insurance, income stability, and social inclusion. Health insurance coverage is scored as 1 for those with government-funded, private, or self-purchased insurance, and 0 for individuals without insurance or those reliant solely on government assistance, illustrating the pivotal role of insurance in accessing medical services. Educational attainment is classified such that individuals with at least a bachelor’s degree score 1, while those with lower educational levels score 0, emphasizing the impact of education on health literacy and lifestyle choices. Access to hospital services is contingent upon geographical and logistical barriers to healthcare; individuals not facing such barriers score 1, while those impeded by transportation or geographic obstacles score 0, underlining the importance of timely access to medical care. Individuals cohabiting with others score 1, while those living alone score 0, reflecting the vital role of social support networks in enhancing emotional well-being and managing health. Depression is assessed using the CES-D scale, with individuals scoring above 10 classified as depressed (score 0), and those scoring below 10 indicating mild or no depressive symptoms (score 1), highlighting the substantial impact of depression on physical health and adherence to treatment. Social participation, measured by engagement in social, recreational, or community activities, assigns a score of 1 to those who participate regularly and 0 to those who do not, demonstrating the positive effects of social engagement in reducing loneliness and enhancing both physical and mental health. Finally, marital status is scored as 1 for married or partnered individuals, and 0 for those who are separated, divorced, widowed, or never married, reflecting the influence of marital status on emotional support and overall well-being. In summary, these SDHs indicators provide a detailed and comprehensive framework for understanding the factors influencing health outcomes, particularly among the elderly population in China. A detailed distribution of SDHs is presented in Supplementary Table 1.

In this longitudinal study, we developed a composite SDHs score to capture the varying degrees of association between different SDHs and long-term health outcomes. This approach, commonly employed in epidemiological research, assesses the cumulative effects of socioeconomic and health-related factors over time [22]. Each SDHs is categorized into two levels: advantaged (scoring 0) and disadvantaged (scoring 1). Focusing on hip fracture risk, we employed Cox regression models to evaluate the cumulative impact of these SDHs over time. The SDHs scores in CHARLS range from 0 to 10, with higher scores indicating more favorable SDHs characteristics. Participants were divided into two groups: one representing individuals with favorable SDHs (score ≥ 7) and the other representing those with disadvantaged SDHs (score < 7). This classification allowed us to examine long-term trends and outcomes associated with different SDHs configurations.

Metabolic syndrome is defined by the presence of ≥ 3 components: hypertension (systolic blood pressure ≥ 130 mmHg, diastolic blood pressure ≥ 85 mmHg) or the use of antihypertensive medications, increased waist circumference (≥ 85 cm in males, ≥ 80 cm in females), elevated low-density lipoprotein (LDL) cholesterol (≥ 130 mg/dL) or treatment for high LDL, low high-density lipoprotein (HDL) cholesterol (men < 40 mg/dL, women < 50 mg/dL) or treatment for low HDL, and elevated triglycerides (≥ 150 mg/dL) or treatment for elevated triglycerides. The use of lipid-lowering medications is determined based on self-reported use of pharmacological treatments for dyslipidemia, specifically those prescribed in Western medicine (Supplementary Table 2). CircS is defined by seven components: short sleep duration (< 6 h/day), depression, and the five components that constitute MetS. A score of ≥ 4 denotes the presence of CircS (Supplementary Table 2) [23–25]. 

### Assessment of hip fracture events

The primary objective of this study was to assess the incidence of hip fractures. To this end, participants were asked a self-reported question: “Since your last interview, have you experienced a hip fracture?” Prior to the interview, the interviewer clarified the precise location of the hip bone to ensure that participants had a clear understanding of the definition of a hip fracture and could provide accurate responses. Responses were recorded as either “Yes” or “No.”

### Covariates

Experienced researchers accounted for sociodemographic characteristics and health behavior variables to control for potential confounders. Sociodemographic factors included age, sex, economic stability (e.g., housing, income, and employment status), social support (e.g., marital status, social participation, and living arrangements), healthcare access (e.g., insurance coverage and proximity to medical facilities), and individual health conditions (e.g., depression and educational attainment). Health behavior variables encompassed smoking and alcohol consumption. Additionally, chronic conditions such as hypertension, dyslipidemia, and diabetes, along with medication use (e.g., antihypertensive, anti-dyslipidemic, and anti-diabetic drugs), were considered. Hypertension was diagnosed using the following criteria: systolic blood pressure ≥ 140 mmHg, diastolic blood pressure ≥ 90 mmHg, current antihypertensive medication use, or self-reported physician diagnosis [26]. Dyslipidemia was diagnosed based on self-reported physician diagnosis, current use of lipid-lowering medications, and/or any of the following laboratory values: triglycerides >150 mg/dL, high-density lipoprotein cholesterol (HDL-C) < 40 mg/dL, low-density lipoprotein cholesterol (LDL-C) >160 mg/dL, or total cholesterol >240 mg/dL [27]. Diabetes was diagnosed using the following criteria: fasting blood glucose ≥ 126 mg/dL, glycosylated hemoglobin (HbA1c) ≥ 6.5%, current use of anti-diabetic medications, or self-reported physician diagnosis of diabetes mellitus [28]. Using the 10-item Center for Epidemiologic Studies Depression Scale (CES-D-10), depressive symptoms were assessed, with a score of 10 marking the presence of elevated symptoms [29]. In older Chinese groups, this threshold has been verified and is frequently applied in national surveys such as CHARLS [18, 30, 31].

### Statistical analysis

The continuous variables in this study were found to follow a non-normal distribution. Non-normally distributed data were summarized using medians and interquartile ranges, while categorical data were expressed as frequencies and percentages. Non-normally distributed continuous variables were compared between the two SDHs groups using the Wilcoxon rank-sum test, and categorical variables were analyzed using the chi-square test. Using the Akaike Information Criterion (AIC) to compare model fits helped guide the knot placement in the restricted cubic spline model. Selected for the final analysis, the model with four knots at the 5th, 35th, 65th, and 95th percentiles of the SDHs distribution provided an ideal balance between model fit and simplicity. Given the lack of a standard threshold for SDHs scores in the context of hip fracture risk, we took a data-driven approach. Specifically, RCS analysis was used to analyze the non-linear correlation between SDHs and the incidence of hip fractures. Using the inflection point observed around a score of 7, we split participants into a favorable SDHs group (score ≥ 7) and a disadvantaged group (score < 7) for subsequent categorical analysis. In two separate analyses, Cox regression models were created: one considering SDHs as a continuous variable and the other using a dichotomized SDHs variable (≥ 7vs.<7). The proportional hazards assumption for each model was further evaluated with Schoenfeld residuals, and no significant violations were observed (*P* > 0.05 for all covariates). Using variance inflation factors (VIFs), multicollinearity among all covariates in each model was evaluated, and all VIF values were found to be less than 3, showing no problematic collinearity. When SDHs was modeled as a categorical variable, the ≥ 7 group was used as the reference. Cox proportional hazards models were used to assess the association between SDHs and hip fracture events. Three models were constructed: Model 1 (unadjusted), which assessed the direct effect of SDHs on fracture risk; Model 2, which adjusted for age and sex; and Model 3, which further adjusted for BMI, rheumatoid arthritis, hypertension, dyslipidemia, diabetes, and cardiovascular disease. Kaplan-Meier curves were used to estimate the cumulative incidence of fractures, and the log-rank test was applied to evaluate differences between groups. Sensitivity analyses were conducted to ensure the robustness of the results, including reanalyzing the relationship between SDHs and fracture risk using a dataset with missing values and excluding fractures that occurred within two years of cohort entry to address potential reverse causality. To assess model discrimination, receiver operating characteristic (ROC) curves were plotted, and the area under the curve (AUC) was calculated to quantify model performance. An AUC > 0.6 was considered indicative of good discrimination. Sensitivity, specificity, positive predictive value (PPV), and negative predictive value (NPV) were evaluated for different cutoff points. Missing data were addressed using multiple imputation by chained equations (MICE), implemented via the mice package in R. Assuming missing at random (MAR), imputations were generated based on the observed data structure to estimate plausible values for incomplete entries. The distribution and proportion of missing values across variables are summarized in Supplementary Table 3. Datasets with multiple imputation provided the primary results, which serve as the main inferential basis for this study. All statistical analyses were performed using R version 4.4.1, and a two-sided p-value < 0.05 was considered statistically significant.

## Results

### Demographics and characteristics of the participants

Table 1 presents a detailed summary of the baseline clinical and sociodemographic characteristics of the study cohort, stratified by SDHs category. The study included 11,928 participants, divided into two groups based on their SDHs scores: 8,306 participants with an SDHs score ≥ 7 and 3,622 participants with an SDHs score < 7. The median age of the cohort was 58 years (IQR: 51–65). Participants in the lower SDHs group were significantly older than those in the higher SDHs group (median: 61 years, IQR: 54–69 vs. median: 57 years, IQR: 50–64). The cohort was composed of 47.32% males and 52.68% females, with males more prevalent in the higher SDHs group (51.13%) and females more common in the lower SDHs group (61.43%). Significant differences between the two SDHs groups were observed across a broad range of variables, including age, sex, BMI, systolic blood pressure, education level, alcohol consumption, smoking status, marital status, income, employment status, healthcare insurance coverage, hospital accessibility, and the prevalence of heart disease, hypertension, diabetes, arthritis/rheumatism, circadian rhythm syndrome, metabolic syndrome, depression, and community support (all *P* < 0.001; Table 1). Additionally, diastolic blood pressure and dyslipidemia also differed significantly between the two groups (both *P* < 0.05; Table 1).

**Table 1:** Baseline characteristics and distribution by social determinants of health categories

Characteristic	Overall	Social determinants of health (≥ 7)	Social determinants of health (< 7)	*p*-value^1^
	*N* = 11,928	*N* = 8,306	*N* = 3,622	
Incident of hip fracture, n (%)	301.00 (2.52%)	171.00 (2.06%)	130.00 (3.59%)	< 0.001
Age, Median (Q1, Q3)	58.00 (51.00, 65.00)	57.00 (50.00, 64.00)	61.00 (54.00, 69.00)	< 0.001
Body mass index, Median (Q1, Q3)	23.12 (20.83, 25.78)	23.41 (21.12, 26.03)	22.37 (20.19, 24.99)	< 0.001
Systolic blood pressure, mmHg, Median (Q1, Q3)	127.33 (115.00, 142.00)	126.67 (115.00, 140.67)	128.67 (115.67, 145.67)	< 0.001
Diastolic blood pressure, mmHg, Median (Q1, Q3)	75.00 (67.33, 83.33)	75.00 (67.67, 83.33)	74.33 (67.00, 83.33)	0.012
Social determinants of health, Median (Q1, Q3)	7.00 (6.00, 8.00)	7.00 (7.00, 8.00)	6.00 (5.00, 6.00)	< 0.001
Gender, n (%)				< 0.001
Male	5,644.00 (47.32%)	4,247.00 (51.13%)	1,397.00 (38.57%)	
Female	6,284.00 (52.68%)	4,059.00 (48.87%)	2,225.00 (61.43%)	
Drinking status, n (%)				< 0.001
Never drink	8,412.00 (70.52%)	5,697.00 (68.59%)	2,715.00 (74.96%)	
Currently drinking	2,945.00 (24.69%)	2,243.00 (27.00%)	702.00 (19.38%)	
Ever drunk	571.00 (4.79%)	366.00 (4.41%)	205.00 (5.66%)	
Smoking status, n (%)				< 0.001
Never smoked	7,308.00 (61.27%)	4,934.00 (59.40%)	2,374.00 (65.54%)	
Currently smoking	3,594.00 (30.13%)	2,641.00 (31.80%)	953.00 (26.31%)	
Ever smoked	1,026.00 (8.60%)	731.00 (8.80%)	295.00 (8.14%)	
Heart disease, n (%)				< 0.001
Yes	1,425.00 (11.95%)	878.00 (10.57%)	547.00 (15.10%)	
No	10,503.00 (88.05%)	7,428.00 (89.43%)	3,075.00 (84.90%)	
Hypertension, n (%)				< 0.001
Yes	4,672.00 (39.17%)	3,087.00 (37.17%)	1,585.00 (43.76%)	
No	7,256.00 (60.83%)	5,219.00 (62.83%)	2,037.00 (56.24%)	
Hyperlipidemia, n (%)				0.038
Yes	1,152.00 (9.66%)	833.00 (10.03%)	319.00 (8.81%)	
No	10,776.00 (90.34%)	7,473.00 (89.97%)	3,303.00 (91.19%)	
Diabetes mellitus, n (%)				0.431
Yes	1,420.00 (11.90%)	976.00 (11.75%)	444.00 (12.26%)	
No	10,508.00 (88.10%)	7,330.00 (88.25%)	3,178.00 (87.74%)	
Arthritis or rheumatism, n (%)				< 0.001
Yes	3,837.00 (32.17%)	2,290.00 (27.57%)	1,547.00 (42.71%)	
No	8,091.00 (67.83%)	6,016.00 (72.43%)	2,075.00 (57.29%)	
Circadian rhythm syndrome, n (%)				< 0.001
Yes	1,753.00 (14.70%)	1,003.00 (12.08%)	750.00 (20.71%)	
No	10,175.00 (85.30%)	7,303.00 (87.92%)	2,872.00 (79.29%)	
Metabolic syndrome, n(%)				
Yes	2,660.00 (22.30%)	1,934.00(23.28%)	726.00 (20.04%)	< 0.001
No	9,268.00 (77.70%)	6.372.00(76.72%)	2.896.00 (79.96%)	
House ownership, n (%)				< 0.001
0	308.00 (2.58%)	104.00 (1.25%)	204.00 (5.63%)	
1	11,620.00 (97.42%)	8,202.00 (98.75%)	3,418.00 (94.37%)	
Income, n (%)				< 0.001
0	9,906.00 (83.05%)	6,394.00 (76.98%)	3,512.00 (96.96%)	
1	2,022.00 (16.95%)	1,912.00 (23.02%)	110.00 (3.04%)	
Employment status, n (%)				< 0.001
0	435.00 (3.65%)	134.00 (1.61%)	301.00 (8.31%)	
1	11,493.00 (96.35%)	8,172.00 (98.39%)	3,321.00 (91.69%)	
Health insurance, n (%)				< 0.001
0	776.00 (6.51%)	191.00 (2.30%)	585.00 (16.15%)	
1	11,152.00 (93.49%)	8,115.00 (97.70%)	3,037.00 (83.85%)	
Education, n (%)				< 0.001
0	11,614.00 (97.37%)	7,999.00 (96.30%)	3,615.00 (99.81%)	
1	314.00 (2.63%)	307.00 (3.70%)	7.00 (0.19%)	
Hospital access, n (%)				< 0.001
0	188.00 (1.58%)	12.00 (0.14%)	176.00 (4.86%)	
1	11,740.00 (98.42%)	8,294.00 (99.86%)	3,446.00 (95.14%)	
Living alone, n (%)				< 0.001
0	1,015.00 (8.51%)	188.00 (2.26%)	827.00 (22.83%)	
1	10,913.00 (91.49%)	8,118.00 (97.74%)	2,795.00 (77.17%)	
Depression, n (%)				< 0.001
0	3,119.00 (26.15%)	710.00 (8.55%)	2,409.00 (66.51%)	
1	8,809.00 (73.85%)	7,596.00 (91.45%)	1,213.00 (33.49%)	
Community surpportation, n (%)				< 0.001
0	7,560.00 (63.38%)	4,453.00 (53.61%)	3,107.00 (85.78%)	
1	4,368.00 (36.62%)	3,853.00 (46.39%)	515.00 (14.22%)	
Marital status, n (%)				< 0.001
0	1,448.00 (12.14%)	218.00 (2.62%)	1,230.00 (33.96%)	
1	10,480.00 (87.86%)	8,088.00 (97.38%)	2,392.00 (66.04%)	

^***1***^Pearson’s Chi-squared test; Wilcoxon rank sum test

## Associations between baseline SDHs scores and incident hip fracture

During the study period, 301 participants (2.52% of the cohort) experienced first hip fracture events. The median follow-up duration was 8.72 years, resulting in a total of approximately 103,999 person-years of observation (11,928 participants×8.72 years). The incidence of hip fractures was significantly higher in participants with lower SDHs scores (< 7) compared to those with higher SDHs scores (≥ 7) (3.59%vs.2.06%, *P* < 0.001; Table 1). Cox proportional hazards regression analysis revealed a significant negative association between SDHs scores and hip fracture risk. In the unadjusted model (Model I), each one-unit increase in SDHs score was associated with a 22% reduction in hip fracture risk (HR = 0.78, 95%CI: 0.71–0.86, *P* < 0.001; Table 2). After adjusting for age and sex (Model II), this association remained significant, with each one-unit increase in SDHs score associated with an 18% reduction in hip fracture risk (HR = 0.82, 95%CI: 0.74–0.91, *P* < 0.001; Table 2). Further adjustments for BMI, arthritis/rheumatism, hypertension, dyslipidemia, diabetes, heart disease, smoking status, and alcohol consumption (Model III) attenuated this association, but each one-unit increase in SDHs score was still associated with a 14% reduction in hip fracture risk (HR = 0.86, 95%CI: 0.77–0.95, *P* = 0.003; Table 2).

**Table 2:** The association between social determinants of health and hip fracture

Characteristic	*N*	Event *N*	Model 1		Model 2		Model 3	
			HR (95%CI)	*p*-value	HR (95%CI)	*p*-value	HR (95%CI)	*p*-value
Social determinants of health (Continuous)	11,928	301	0.78 (0.71, 0.86)	< 0.001	0.82 (0.74, 0.91)	< 0.001	0.86 (0.77, 0.95)	0.003
Social determinants of health (Category)	11,928	301						
Social determinants of health(≥ 7)	8,306	171	Reference	Reference	Reference	Reference	Reference	Reference
Social determinants of health(< 7)	3,622	130	1.76 (1.40, 2.21)	< 0.001	1.55 (1.22, 1.96)	< 0.001	1.41 (1.11,1.80)	0.005

Model1: unadjusted; Model 2: adjusted for age, gender; Model 3: Model 2 + adjusted for smoking status, drinking status, BMI, arthritis or rheumatism, hypertension, hyperlipidemia, diabetes mellitus, heart disease, CircS

*Abbreviation:* *HR* Hazard ratio, *CI* Confidence interval, *CircS* Circadian rhythm syndrome

To further explore this relationship, we categorized SDHs scores into two groups (≥ 7vs.<7). In the unadjusted model (Model I), participants with lower SDHs scores (< 7) had a 76% higher risk of hip fractures compared to those with higher SDHs scores (≥ 7) (HR = 1.76, 95%CI: 1.40–2.21, *P* < 0.001; Table 2). After adjusting for age and sex (Model II), the risk remained significantly elevated, with the lower SDHs group showing a 55% higher risk of hip fractures (HR = 1.55, 95%CI: 1.22–1.96, *P* < 0.001; Table 2). After further adjustment for BMI, arthritis/rheumatism, hypertension, dyslipidemia, diabetes, heart disease, smoking status, and alcohol consumption (Model III), the association was attenuated, but the lower SDHs group still exhibited a 41% higher risk of hip fractures (HR = 1.41, 95%CI: 1.10–1.81, *P* = 0.005; Table 2).

**Fig. 2:**
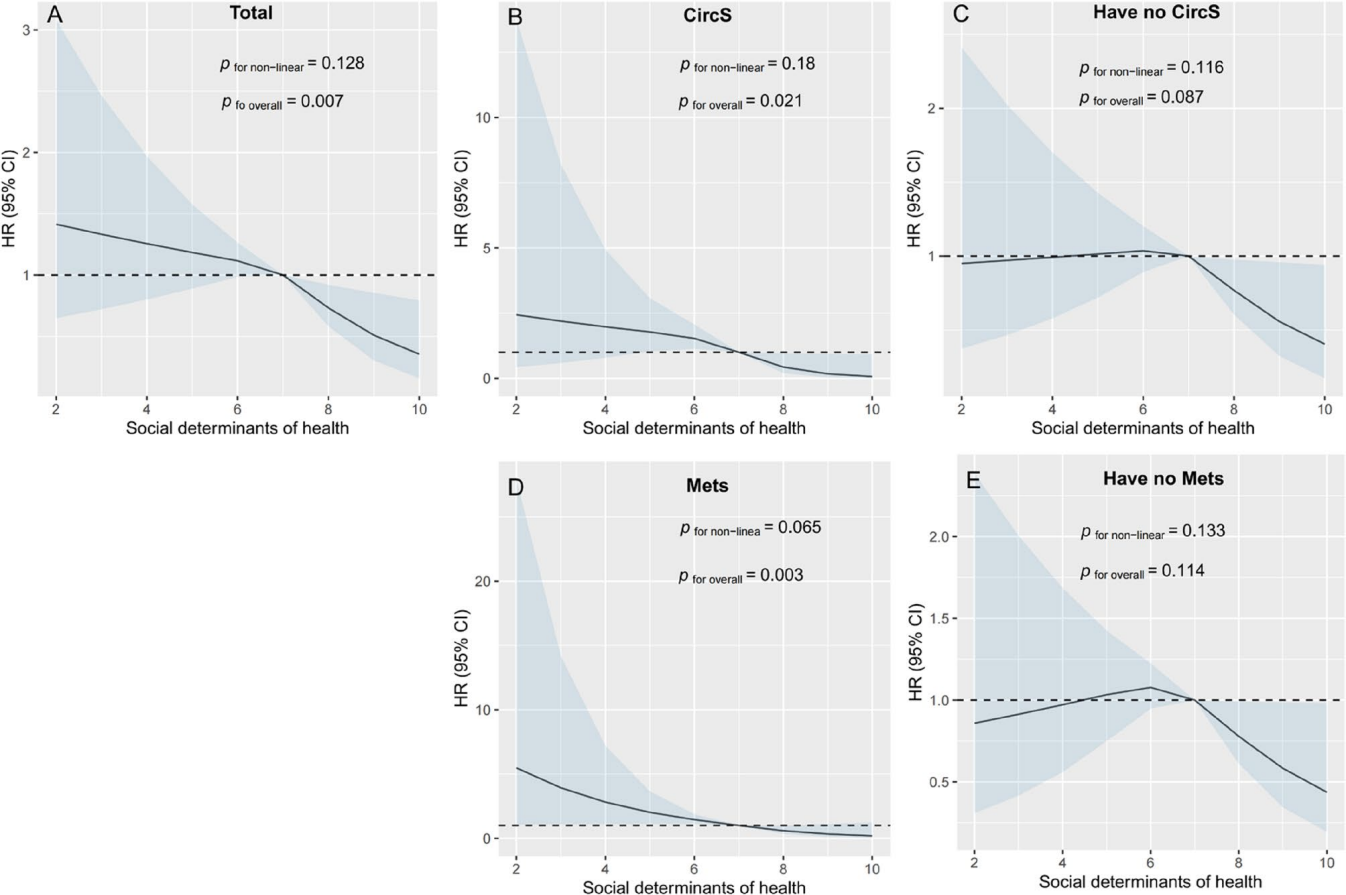
Association of SDHs and the risk of hip fracture using a multivariable-adjusted restricted cubic spines model. Restricted cubic spline analysis has four knots at the 5th, 35th, 65th, and 95th percentiles of SDHs. **A** total participants; (**B)** participants with CircS; (**C)** participants with no CircS; (**D)** participants with MetS; (**E)** participants with no MetS. Abbreviation: SDHs, social determinants of health; CircS, circadian rhythm syndrome; MetS, metabolic syndrome; HR, Hazard Ratio; CI, Confidence Interval

**Fig. 3:**
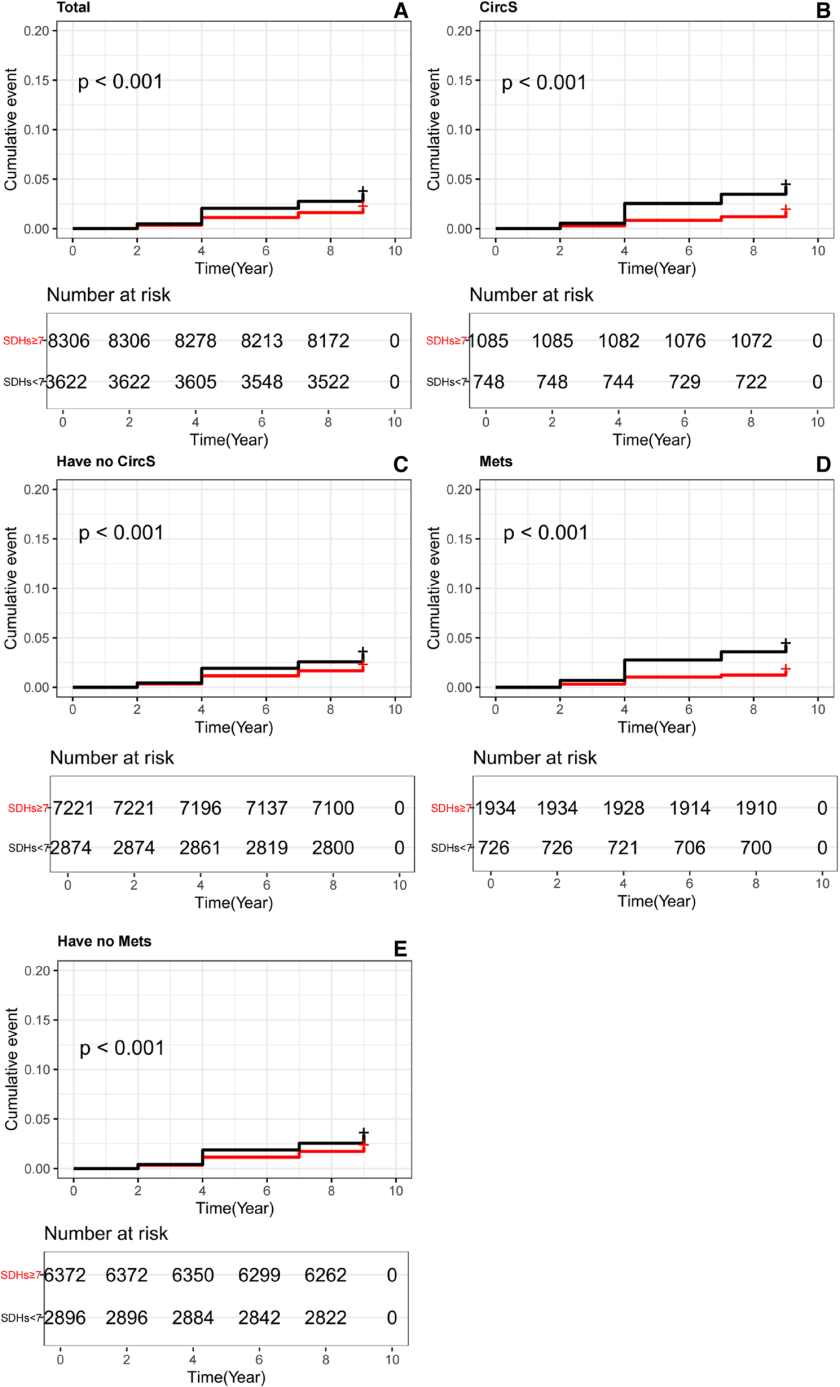
The Kaplan-Meier analysis for hip fracture was based on SDHs quartiles for total participants (**A)**, participants with CircS (**B)**, participants with no CircS (**C)**, participants with MetS (**D)** and participants with no MetS (**E)**. Abbreviation: SDHs, social determinants of health; CircS, circadian rhythm syndrome; MetS, metabolic syndrome

Figure 2A presents the dose-response curve from the multivariable-adjusted RCS model, which depicts the relationship between SDHs scores and hip fracture risk. The curve demonstrates a strong linear association, indicating that the risk of hip fractures decreases progressively as SDHs scores increase. This trend was consistent after adjusting for various covariates in the overall population (overall *P* = 0.007; non-linear *P* = 0.128). Fig. 3A displays the Kaplan-Meier survival curves, stratified by SDHs scores, showing the cumulative probability of surviving without hip fractures in the overall population (≥ 7vs.<7). Participants with lower SDHs scores (< 7) had a significantly lower survival probability free of hip fractures compared to those with higher SDHs scores (≥ 7) (log-rank test *P* < 0.001).

### Associations between SDHs scores and hip fracture risk stratified by metabolic and circadian rhythm States

During the follow-up period, 63 participants with MetS and 238 participants without MetS experienced hip fractures. Similarly, 57 participants with CircS and 244 participants without CircS experienced hip fractures. To further examine the relationship between SDHs scores and hip fracture risk, participants were stratified according to the presence of MetS and CircS. Among participants with MetS, the fully adjusted model (Model III) revealed that each one-unit increase in SDHs score was associated with a 33% reduction in hip fracture risk (HR = 0.67, 95%CI: 0.53–0.84, *P* < 0.001; Table 3). Furthermore, participants with lower SDHs scores (< 7) had a 2.39-fold increased risk of hip fractures compared to those with higher SDHs scores (≥ 7) (HR = 2.39, 95%CI: 1.42–4.01, *P* < 0.001; Table 3). In contrast, among participants without MetS, each one-unit increase in SDHs score was associated with a 9% reduction in hip fracture risk, though this association did not reach statistical significance in the fully adjusted model (HR = 0.91, 95%CI: 0.81–1.03, *P* = 0.123; Table 3). In this subgroup, participants with lower SDHs scores (< 7) had a 1.22-fold higher risk of hip fractures compared to those with higher SDHs scores (≥ 7), but this association also lacked statistical significance (HR = 1.22, 95%CI: 0.93–1.60, *P* = 0.151; Table 3). Among participants with CircS, each one-unit increase in SDHs score was associated with a 29% reduction in hip fracture risk in the fully adjusted model (HR = 0.71, 95%CI: 0.56–0.90, *P* = 0.005; Table 3). Additionally, compared to participants with higher SDHs scores (≥ 7), those with lower SDHs scores (< 7) had a 2.62-fold increased risk of hip fractures (HR = 2.62, 95%CI: 1.46–4.71, *P* = 0.001; Table 3). In contrast, among participants without CircS, each one-unit increase in SDHs score was associated with a 10% reduction in hip fracture risk, although this association was not statistically significant in the fully adjusted model (HR = 0.90, 95%CI: 0.80–1.02, *P* = 0.092; Table 3). In this subgroup, participants with lower SDHs scores (< 7) had a 1.18-fold higher risk of hip fractures compared to those with higher SDHs scores (≥ 7), but this association was also not statistically significant (HR = 1.18, 95%CI: 0.90–1.55, *P* = 0.235; Table 3).

**Table 3:** The association between social determinants of health and hip fracture according to varying metabolic and circadian rhythm conditions

Characteristic	*N*	Event *N*	Model 1		Model 2		Model 3	
			HR (95%CI)	*p*-value	HR (95%CI)	*p*-value	HR (95%CI)	*p*-value
MetS								
Social determinants of health (Continuous)	2,660	63	0.66 (0.54, 0.81)	< 0.001	0.66 (0.53, 0.82)	< 0.001	0.67 (0.53, 0.84)	< 0.001
Social determinants of health (Category)	2,660	63						
Social determinants of health(≥ 7)	1,934	32	Reference	Reference	Reference	Reference	Reference	Reference
Social determinants of health(< 7)	726	31	2.62 (1.60, 4.29)	< 0.001	2.60 (1.56, 4.31)	< 0.001	2.39 (1.42, 4.01)	< 0.001
No-MetS								
Social determinants of health (Continuous)	9,268	238	0.81 (0.73, 0.91)	< 0.001	0.88 (0.78, 0.98)	0.026	0.91 (0.81, 1.03)	0.123
Social determinants of health (Category)	9,268	238						
Social determinants of health(≥ 7)	6,372	139	Reference	Reference	Reference	Reference	Reference	Reference
Social determinants of health(< 7)	2,896	99	1.58 (1.22, 2.04)	< 0.001	1.33 (1.02, 1.75)	0.036	1.22 (0.93, 1.60)	0.151
CircS								
Social determinants of health (Continuous)	1,753	152	0.68 (0.55, 0.84)	< 0.001	0.69 (0.55, 0.85)	< 0.001	0.71 (0.56, 0.90)	0.005
Social determinants of health (Category)	1,753	152						
Social determinants of health(≥ 7)	1,003	57	Reference	Reference	Reference	Reference	Reference	Reference
Social determinants of health(< 7)	750	95	3.21 (1.82, 5.67)	< 0.001	3.07 (1.72, 5.45)	< 0.001	2.62 (1.46, 4.71)	0.001
No-CircS								
Social determinants of health (Continuous)	10,175	44	0.82 (0.73, 0.91)	< 0.001	0.87 (0.77, 0.97)	0.015	0.90 (0.80, 1.02)	0.092
Social determinants of health (Category)	10,175	44						
Social determinants of health(≥ 7)	7,303	19	Reference	Reference	Reference	Reference	Reference	Reference
Social determinants of health(< 7)	2,872	25	1.49 (1.15, 1.93)	0.003	1.30 (0.99, 1.70)	0.060	1.18 (0.90, 1.55)	0.235

Model1: unadjusted; Model 2: adjusted for age, gender; Model 3: Model 2 + adjusted for smoking status, drinking status, BMI, arthritis or rheumatism, hypertension, hyperlipidemia, diabetes mellitus, heart disease

*Abbreviation*: *HR* Hazard ratio, *CI* Confidence interval, *MetS* Metabolic syndrome, *CircS* Circadian rhythm syndrome

Figure 2B and E show the RCS curves, demonstrating a strong linear relationship between SDHs scores and hip fracture risk in subgroups stratified by CircS and MetS status. This linear trend was particularly pronounced in participants with MetS and CircS, where higher SDHs scores were associated with progressively lower hip fracture risk. Fig. 3B and E depict the Kaplan-Meier survival curves, illustrating the cumulative probability of surviving without hip fractures, stratified by SDHs scores (≥ 7 vs.<7) and further categorized by CircS and MetS status. Compared to participants with higher SDHs scores (≥ 7), those with lower SDHs scores (< 7) exhibited a significantly lower survival probability free of hip fractures (log-rank test *P* < 0.001). This pattern was especially evident among participants with comorbidities such as CircS or MetS, where lower SDHs scores were associated with the lowest survival probability free of hip fractures.

### Subgroup analyses

The subgroup analysis demonstrated that higher SDHs scores were significantly associated with a reduced risk of hip fractures across several subgroups. In women, higher SDHs scores were linked to a 15% reduction in risk (HR = 0.85, *P* = 0.021). Similarly, both older participants (≥ 60 years: HR = 0.86, *P* = 0.041) and younger participants (< 60 years: HR = 0.85, *P* = 0.033) exhibited significant reductions in hip fracture risk. Among participants who never drank alcohol, higher SDHs scores were associated with a significantly lower risk of hip fractures (HR = 0.83, *P* = 0.003), as was the case for those who never smoked (HR = 0.86, *P* = 0.021). Additionally, participants with a BMI < 28 (HR = 0.88, *P* = 0.024) and those without arthritis/rheumatism (HR = 0.77, *P* < 0.001) demonstrated a significant reduction in risk with higher SDHs scores. No significant associations were observed in men, current or former alcohol drinkers, current or former smokers, participants with a BMI ≥ 28, or those with arthritis/rheumatism (Supplementary Table 4). Nonetheless, no statistically significant interactions were found among these subgroups (all Pinteraction > 0.05; Supplementary Table 4), indicating that the apparent differences between subgroups should be interpreted with caution.

Further analysis using categorized SDHs scores (< 7vs.≥7) revealed that lower SDHs scores were significantly associated with an increased risk of hip fractures in several subgroups. Women with lower SDHs scores had a 37% higher risk (HR = 1.37, *P* = 0.040), while participants aged ≥ 60 years had a 62% higher risk (HR = 1.62, *P* = 0.004). Among participants who never drank alcohol, those with lower SDHs scores had a 51% higher risk (HR = 1.51, *P* = 0.004), and current smokers had a 72% higher risk (HR = 1.72, *P* = 0.020). Furthermore, participants with a BMI < 28 (HR = 1.34, *P* = 0.024) and those without arthritis/rheumatism (HR = 1.63, *P* = 0.004) showed a significantly higher risk of hip fractures with lower SDHs scores. No significant associations were found in men, younger participants (< 60 years), current or former alcohol drinkers, former or never smokers, participants with a BMI ≥ 28, or those with arthritis/rheumatism (Supplementary Table 5).

### Sensitivity analysis

In this analysis, we excluded the dataset with missing values and presented the results from Cox regression to evaluate the association between SDHs and hip fracture risk. Both unadjusted and adjusted models demonstrated a significant positive correlation between SDHs and hip fracture risk, with the association remaining robust even after adjusting for various confounding factors (Supplementary Table 6). Consistent with the results in Table 2, the analysis confirmed that excluding missing data did not substantially alter the findings. This suggests that the multiple imputation method effectively addressed the issue of missing data, thereby strengthening the robustness of the study’s conclusions. Specifically, the results from the imputed dataset were highly consistent with those from the complete case analysis, underscoring the appropriateness and reliability of the imputation method for handling missing data. A sensitivity analysis excluding hip fractures occurring within the first two years of follow-up continued to show a significant negative correlation between SDHs scores and hip fracture risk. After adjusting for confounders, each one-unit increase in SDHs score was associated with a reduction in hip fracture risk (Supplementary Table 7). Furthermore, after adjusting for confounders, participants with lower SDHs scores (< 7) exhibited a significantly higher risk of hip fractures compared to those with higher SDHs scores (≥ 7) (Supplementary Table 7). These findings underscore the robustness of the association between SDHs scores and hip fracture risk, even when early fracture events were excluded.

### The predictive value of baseline SDHs for hip fracture risk

**Fig. 4:**
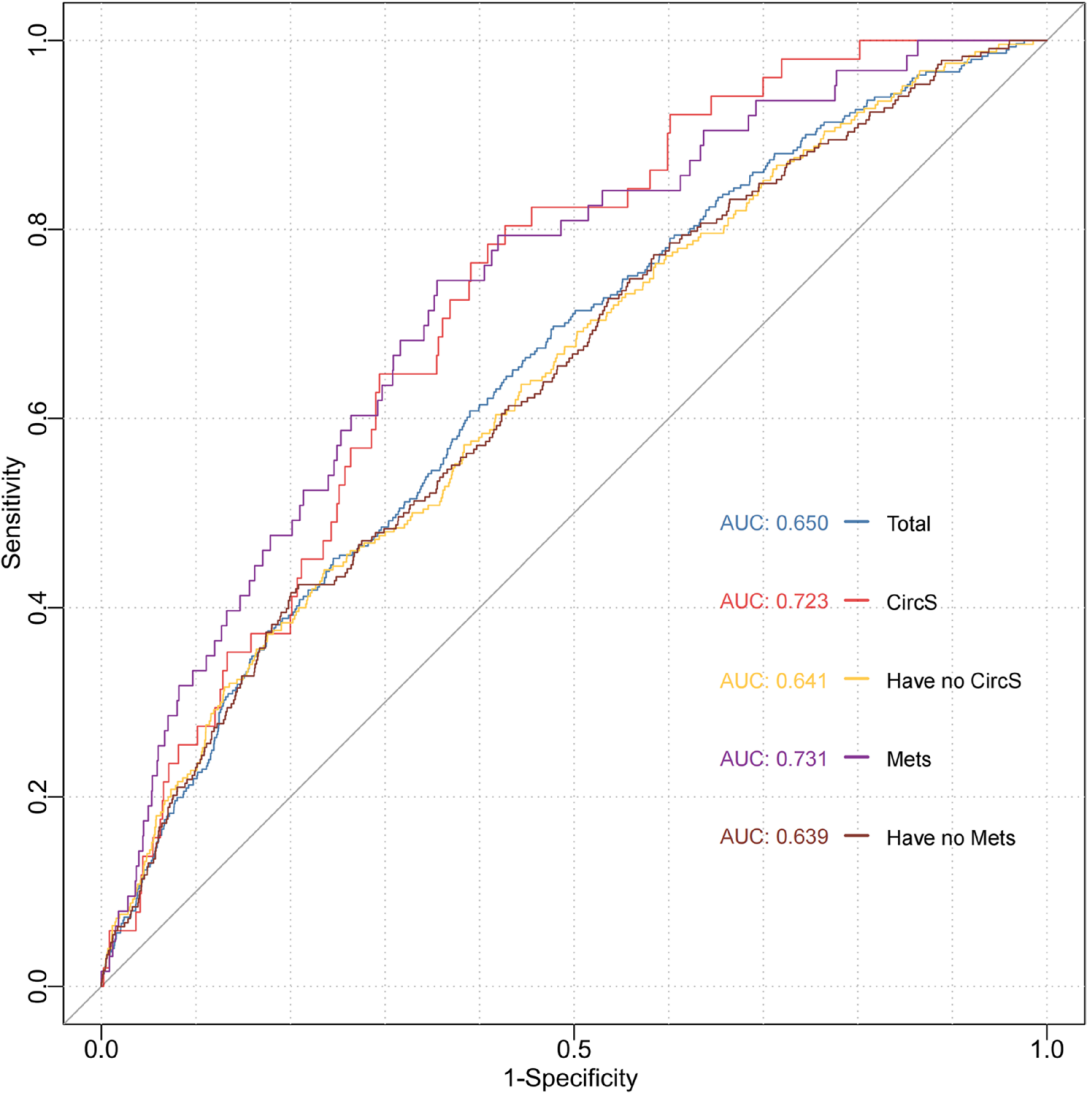
ROC Curves for SDHs in predicting hip fracture risk. Abbreviation: SDHs, social determinants of health; CircS, circadian rhythm syndrome; MetS, metabolic syndrome; ROC, receiver operating characteristic curve; AUC, area under the curve

To evaluate the predictive value of SDHs for hip fracture risk, we performed ROC analysis and calculated the AUC. The ROC analysis revealed that SDHs scores demonstrated moderate to strong predictive power for hip fracture risk across different subgroups. In the overall population, the AUC was 0.650 (95%CI: 0.620–0.681, *P* < 0.001), indicating moderate predictive ability (Figs. 4). Among participants with CircS, the AUC was 0.723 (95%CI: 0.663–0.783, *P* < 0.001), reflecting stronger predictive performance, while for those without CircS, the AUC was 0.641 (95%CI: 0.606–0.676, *P* < 0.001) (Figs. 4). Similarly, participants with MetS showed a strong predictive ability (AUC = 0.731, 95%CI: 0.671–0.791, *P* < 0.001), whereas those without MetS had moderate predictive ability (AUC = 0.639, 95%CI: 0.604–0.675, *P* < 0.001) (Figs. 4). Different subgroups had varying optimal thresholds for SDHs scores (Supplementary Table 8). In the group with CircS, the model’s sensitivity was highest at 0.804 (95%CI: 0.695–0.913), with a specificity of 0.573 (95%CI: 0.550–0.596). The corresponding PPV and NPV were 0.051 (95%CI: 0.036–0.066) and 0.990 (95%CI: 0.984–0.996), respectively. Likewise, among those with MetS, the sensitivity was 0.746 (95%CI: 0.639–0.854) and the specificity was 0.645 (95%CI: 0.627–0.663), with a PPV of 0.049 (95%CI: 0.035–0.062) and an NPV of 0.991 (95%CI: 0.986–0.995). These findings emphasize the importance of considering social determinants of health in predicting hip fracture risk, particularly in individuals with metabolic and circadian rhythm disorders.

## Discussion

This longitudinal study, utilizing CHARLS data, underscores the pivotal role of SDHs in reducing the risk of hip fractures among Chinese adults aged 45 and older. The study examines how SDHs influence hip fracture risk, with particular emphasis on how MetS and CircS modulate this relationship. The findings reveal a significant negative correlation between SDHs scores and hip fracture risk, with higher SDHs scores being associated with a lower risk of hip fractures. Participants with SDHs scores below 7 exhibited a notably higher risk of hip fractures compared to those with scores of 7 or greater. These associations remained robust even after adjusting for multiple confounding factors, with the relationship being further strengthened in individuals with MetS and CircS. Sensitivity analyses affirmed the consistency of these findings, even after excluding early fracture events. Furthermore, SDHs demonstrated moderate to strong predictive value for hip fracture risk, particularly in individuals with MetS and CircS. These results highlight the critical importance of incorporating SDHs into fracture risk assessments, especially in individuals with these comorbidities.

Our findings both resonate with and significantly extend previous research emphasizing the pivotal role of SDHs in shaping health outcomes, particularly in aging populations. Similar to the work of Auais et al., which highlighted the impact of social factors such as socioeconomic status and social support on recovery following hip fractures, and Robles et al., who explored the broader influence of SDHs on healthy aging, our study confirms that higher SDHs scores are associated with a reduced risk of hip fractures [1, 2]. However, our research diverges by integrating metabolic and circadian rhythm factors into the analysis, offering a more nuanced understanding of how these elements interact with SDHs. While Zhang et al. and Carnovali et al. examined the effects of metabolic syndrome and circadian rhythm disorders on bone health, respectively, our study uniquely combines these factors with SDHs, revealing the particularly protective role of higher SDHs scores in individuals with metabolic syndrome MetS and CircS [3, 4]. This finding aligns with the work of Shi et al., who suggested that circadian rhythm disorders may be a stronger predictor of cardiovascular diseases than metabolic syndrome, highlighting the broader implications of circadian disruption on various health outcomes, including bone health [23]. Moreover, our results align with the observations of Lopes et al. and Rinonapoli et al., who emphasized the complex relationship between metabolic health and bone integrity. However, our study expands upon these findings by demonstrating how social factors can mitigate these risks [5, 6]. Specifically, we found that individuals with higher SDHs scores had a 14% lower risk of hip fractures, with an even more pronounced protective effect observed in those with MetS and CircS. This suggests that addressing social inequalities could be particularly beneficial for individuals with metabolic and circadian rhythm disorders, adding a new dimension to the existing fracture prevention literature. Furthermore, our study builds on the work of Song et al. and Tian et al., who explored the role of circadian rhythms in bone metabolism. Our findings support their assertion that circadian rhythm disruption exacerbates the risk of hip fractures in patients with mild hip osteoarthritis, underscoring the need for integrated interventions that address both social and biological determinants of health [11, 12] By elucidating the interactions between SDHs, metabolic health, and circadian rhythms, our research offers a more comprehensive framework for understanding hip fracture risk, particularly in populations with comorbidities, and provides new directions for targeted interventions that account for the multifaceted nature of health determinants.

The mechanisms underlying the associations between SDHs, MetS, CircS, and hip fracture risk are multifactorial, involving complex interactions across biological, behavioral, environmental, and psychosocial domains. Our findings suggest that higher SDHs scores may reduce hip fracture risk by enhancing access to healthcare, improving nutrition, and strengthening social support networks. These factors collectively contribute to better bone health and a lower risk of falls. Our results align with previous studies showing that socioeconomic factors such as income and educational attainment significantly influence physical activity patterns, which in turn affect bone mineral density and fracture susceptibility [10, 32]. Higher socioeconomic status is often associated with better access to preventive healthcare services, including bone density screenings and fall prevention programs, both of which have been shown to reduce fracture risk.Moreover, the protective effect of higher SDHs scores in individuals with MetS and CircS may be partially explained by the role of metabolic and circadian factors in bone metabolism. For example, insulin resistance and chronic inflammation associated with MetS, as noted by Liu et al., can reduce bone formation and increase bone resorption. However, higher SDHs scores may help mitigate these effects by facilitating more effective management of metabolic conditions and encouraging healthier lifestyle choices [7]. Individuals with higher SDHs scores are more likely to access medications, dietary interventions, and lifestyle modifications that counteract the negative impact of MetS on bone health. Similarly, circadian disruptions, as discussed by Swanson et al., can impair bone remodeling and increase fracture risk. However, individuals with higher SDHs scores tend to maintain more regular sleep patterns and are more likely to benefit from interventions that alleviate circadian disturbances [15]. For example, higher SDHs levels may support consistent sleep schedules, reduce shift work, and increase access to treatments for sleep disorders, all of which contribute to improved circadian rhythms and better bone health. The interaction between SDHs and metabolic/circadian health is also influenced by behavioral factors such as physical activity and smoking cessation, which are more common among individuals with higher SDHs scores. These findings align with previous research linking sedentary lifestyles and physical inactivity, which are more prevalent among individuals with lower socioeconomic status, to negative effects on bone health [9, 33]. Elevated SDHs scores encourage healthier behaviors, including regular exercise and smoking cessation, both of which enhance bone density and reduce fracture risk. Furthermore, psychosocial factors like stress and depression, which are more common among individuals with lower SDHs scores, may exacerbate fracture risk by adversely affecting metabolic health and circadian rhythms. Depression is associated with poor sleep quality, reduced physical activity, and increased inflammation, all of which contribute to poor bone health [29, 34]. Additionally, dietary factors, such as vitamin D and calcium intake, which are critical for bone health, are often influenced by socioeconomic status. Individuals with higher SDHs levels are more likely to maintain adequate nutrient intake, thereby reducing the risk of bone density loss and fracture occurrence [35, 36]. The built environment, including access to safe walking paths and recreational facilities, plays a crucial role in promoting physical activity and reducing fall risk [4, 37]. Social isolation and loneliness, which are more common among individuals with lower SDHs scores, have also been shown to negatively impact both mental and physical health, including bone health [38, 39]. By synthesizing these potential mechanisms, our study provides a comprehensive framework for understanding how SDHs scores interact with metabolic and circadian factors to influence hip fracture risk. This integrative approach offers valuable insights into the biological and social pathways underlying these relationships and underscores the importance of addressing both social and biological determinants of health in fracture prevention, particularly in populations with comorbid metabolic and circadian rhythm disorders.

Although this study offers significant insights into the relationships among SDHs, MetS, CircS, and hip fracture risk, several limitations warrant consideration. First, the reliance on self-reported data for hip fractures and certain covariates, such as physical activity and dietary intake, introduces the potential for recall bias and measurement error, which may compromise the accuracy of the findings. Second, while the CHARLS dataset is nationally representative, the generalizability of our results to populations in other regions or with differing socioeconomic and healthcare systems remains uncertain. Third, the observational design of this study limits the ability to infer causality, as residual confounding from unmeasured variables, such as genetic predisposition and environmental influences, cannot be fully excluded. Fourth, although the composite SDHs score provides a useful measure of cumulative social risk, it may oversimplify the intricate and multidimensional nature of social determinants, potentially obscuring individual-level heterogeneity. Fifth, this study is the inability to account for death as a competing risk due to the lack of access to systematically collected mortality data. In particular, the CHARLS has not been linked to national vital registration or death certification systems, making it impossible to obtain accurate and complete information on participants’ vital status. Sixth, it is worth noting that the ¥9,000 income threshold was determined based on historical quintile data from the National Bureau of Statistics and represents a rounded, conservative estimate. While this classification improves interpretability within the CHARLS dataset, which does not contain official income groupings, it may not fully capture regional or temporal variation in income distribution. Finally, the study did not account for temporal variations in SDHs, metabolic health, or circadian rhythms over the follow-up period, which may have influenced the observed associations.

## Conclusions

Higher SDHs scores were linked to a reduced risk of hip fractures, with a stronger connection observed in those with MetS and CircS. These findings stress the importance of factoring SDHs into fracture risk assessments and prevention efforts, especially for groups at elevated risk. Future studies should further explore SDHs trajectories and validate these results in other populations.

## Supplementary Information

Supplementary Material 1.

Supplementary Material 2.

## Data Availability

The datasets underpinning the findings of this study are publicly accessible in an open-access repository. The CHARLS datasets employed and analyzed in this investigation are available through the official CHARLS website at http://charls.pku.edu.cn/en.

## Abbreviations

CHARLS: China Health and Retirement Longitudinal Study
SDHs: Social Determinants of Health
MetS: Metabolic Syndrome
CircS: Circadian Syndrome
BMI: Body Mass Index
HDL: High-Density Lipoprotein
LDL: Low-Density Lipoprotein
ROC: Receiver Operating Characteristic
AUC: Area Under the Curve
CES-D: Center for Epidemiologic Studies Depression Scale
