# Supplementary material for: Exploring social determinants of health in the context of metabolic and circadian influences on new hip fracture risk: longitudinal insights from CHARLS
Source: BMC Public Health. 2025 Nov 21;25:4074. doi: 10.1186/s12889-025-25126-5 (PMC12639743; doi:10.1186/s12889-025-25126-5)
Supplement: Supplementary file 1 — Supplementary Material 1. [file 12889_2025_25126_MOESM1_ESM.docx]

**Supplementary Table 1** Components and scoring of the social determinants of health index

| **Social determinants of health** | **Description** | **Score** |
| --- | --- | --- |
| **House ownership** | Is the house you currently reside in rented? If yes, it indicates that the house is being rented; if no, it suggests the house is owned. | 0: points for renting;  1: point for owning the house. |
| **Household income** | The average household income per member was 9,000 RMB, derived by summing total earnings from wages, agricultural activities, and private enterprise operations, and dividing this sum by the number of individuals residing in the household. | 0: Less than 9,000 RMB；  1: Average total household income before tax exceeds 9,000 RMB |
| **Employment status** | Have you ever worked? Are you retired? | 0: No employment history or currently unemployed；  1: Retired or currently employed" |
| **Health Insurance** | (1) Urban employee medical insurance (yi-bao).  (2) Urban resident medical insurance.  (3) New cooperative medical insurance (he-zuo-yi-liao).  (4) Urban and rural resident medical insurance.  (5) Government medical insurance (gong-fei).  (6) Medical aid.  (7) Private medical Insurance: Purchased by R’s union.  (8) Private medical Insurance: Purchased by Individual.  (9) Other medical insurance.  (10) No insurance. | 0: Without health insurance or receiving government assistance;  1: Covered by government-funded insurance, private insurance, or self-purchased health plans. |
| **Educational Attainment** | Based on self-reported highest educational attainment. | 0: Less than a bachelor's degree;  1: Bachelor's degree or higher" |
| **Access to Hospital Services** | The participants reported that transportation barriers prevented them from accessing medical care. | 0: Transportation barriers  1: No transportation barriers, with medical facilities in proximity. |
| **Living alone** | Do you currently live alone? Yes/No | 0: Living Alone; 1: Co-living With Others" |
| **Depression** | The CES-D score refers to the Center for Epidemiologic Studies Depression Scale (CES-D), a tool designed to assess the severity of depressive symptoms. | 0: CES-D score exceeds 10;  1: Does not exceed 10. |
| **Social Activities** | In the past month, how often have you engaged in these activities (volunteer or charitable activities, caring for patients or disabled individuals, helping family members, friends, or neighbors, attending educational or training courses, socializing with friends, engaging in sports or recreational activities, participating in social or other types of clubs, attending organizational activities, trading stocks, or browsing the internet)? Was it nearly every day, nearly every week, or not frequently? | 0: All activities are 'not frequent';  1: Participates frequently in at least one activity. |
| **Marital Status** | What is your current marital status? | 0: Separated, divorced, widowed, or never married；  1: married and cohabiting, or in a Long-term Partnership. |

**Supplementary Table 2** Definition of metabolic syndrome and circadian rhythm syndrome

| **Measure** | **Categorical cut points** | **Included in MetS** | **Included in CircS** |
| --- | --- | --- | --- |
| Elevated waist circumference | Population- and country-specific definitions (≥85 cm in men, ≥80 in women) | ✔ | ✔ |
| Elevated triglycerides  (drug treatment for elevated triglycerides is an alternate indicator) | ≥150 mg dL^-1^ (1.7 mmol L^-1^) | ✔ | ✔ |
| Reduced HDL-C  (drug treatment for reduced HDL-C is an alternate indicator） | <40 mg dL^-1^ (1.0 mmol L^-1^) in men; <50 mg dL^-1^ (1.3 mmol L^-1^) in women | ✔ | ✔ |
| Elevated blood pressure  (antihypertensive drug treatment in a patient with a history of hypertension is an alternate indicator) | Systolic ≥ 135 and/or  diastolic ≥ 85 mmHg | ✔ | ✔ |
| Elevated fasting glucose  (drug treatment of elevated glucose is an alternate indicator) | ≥100 mg dL^-1^ | ✔ | ✔ |
| Short sleep | <6 hours day^-1^ |  | ✔ |
| Depression symptom | 10 item CES-D score ≥ 10 |  | ✔ |
| **Definition criteria** |  | **≥3 components** | **≥4 components** |

**Abbreviation：**HDL-C, high-density lipoprotein cholesterol; MetS; Metabolic syndrome; CircS: circadian rhythm syndrome

**Supplementary Table 3** Distribution of missing data

| **Characteristic** | **No. of missing values** | **Percent(%)** |
| --- | --- | --- |
| **Incident of hip fracture** | 0 | 0.00 |
| **Age** | 0 | 0.00 |
| **Gender** | 9 | 0.08 |
| **Drinking status** | 2 | 0.02 |
| **Smoking status** | 1 | 0.01 |
| **Body Mass Index** | 1,879 | 15.75 |
| **Systolic Blood Pressure** | 1,814 | 15.21 |
| **Diastolic Blood Pressure** | 1,831 | 15.35 |
| **Heart Disease** | 38 | 0.32 |
| **Hypertension** | 3 | 0.03 |
| **Hyperlipidemia** | 127 | 1.06 |
| **Diabetes mellitus** | 22 | 0.18 |
| **Arthritis or rheumatism** | 17 | 0.14 |
| **Circadian rhythm syndrome** | 5,486 | 45.99 |
| **Social determinants of health** | 0 | 0.00 |
| Income | 0 | 0.00 |
| Education | 0 | 0.00 |
| Depression | 0 | 0.00 |
| Living alone | 0 | 0.00 |
| Marital Status | 0 | 0.00 |
| Hospital access | 0 | 0.00 |
| Health insurance | 0 | 0.00 |
| House ownership | 0 | 0.00 |
| Employment status | 0 | 0.00 |
| Community surpportation | 0 | 0.00 |

**Supplementary Table 4** Subgroup analysis of the association between social determinants of health (continuous) ) and hip fracture

| **Characteristic** | **N** | **Event N** | **HR (95%CI)** | **p-value** | **Pinteraction** |
| --- | --- | --- | --- | --- | --- |
| **Gender** |  |  |  |  | 0.974 |
| Male | 5,643 | 111 | 0.88 (0.74, 1.03) | 0.119 |  |
| Female | 6,285 | 190 | 0.85 (0.75, 0.98) | 0.021 |  |
| **Age** |  |  |  |  | 0.852 |
| ≥60 y | 5,238 | 154 | 0.86 (0.75, 0.99) | 0.041 |  |
| <60 y | 6,690 | 147 | 0.85 (0.73, 0.99) | 0.033 |  |
| **Drinking status** |  |  |  |  | 0.440 |
| Never drink | 8,412 | 221 | 0.83 (0.73, 0.94) | 0.003 |  |
| Currently drinking | 2,945 | 66 | 0.94 (0.75, 1.18) | 0.604 |  |
| Ever drunk | 571 | 14 | 0.90 (0.55, 1.49) | 0.691 |  |
| **Smoking status** |  |  |  |  | 0.662 |
| Never smoked | 7,308 | 202 | 0.86 (0.75, 0.98) | 0.021 |  |
| Currently smoking | 3,594 | 82 | 0.87 (0.71, 1.05) | 0.144 |  |
| Ever smoked | 1,026 | 17 | 0.80 (0.53, 1.19) | 0.267 |  |
| BMI |  |  |  |  | 0.057 |
| BMI≥28 | 1,383 | 30 | 0.69 (0.50, 0.95) |  |  |
| BMI<28 | 10,545 | 271 | 0.88 (0.79, 0.98) | 0.024 |  |
| **Arthritis or rheumatism** |  |  |  |  | 0.058 |
| Yes | 3,836 | 139 | 0.96 (0.82,1.12) | 0.616 |  |
| No | 8,092 | 162 | 0.77 (0.67, 0.89) | <0.001 |  |

**Model1:** unadjusted; **Model 2:** adjusted for age, gender**; Model 3:** Model 2+adjusted for smoking status, drinking status, BMI, arthritis or rheumatism, hypertension, hyperlipidemia, diabetes mellitus, heart disease, CircS

**Abbreviation:** HR, hazard ratio; CI, confidence interval; CircS, circadian rhythm syndrome

**Supplementary Table 5 Subgroup analysis of the association between social determinants of health (Category) and hip fracture**

| **Characteristic** | **N** | **Event N** | **HR (95%CI)** | **p-value** |
| --- | --- | --- | --- | --- |
| **Male** | 5,643 | 111 |  |  |
| Social determinants of health (≥7) | 4,247 | 71 | Reference | Reference |
| Social determinants of health (<7) | 1,396 | 40 | 1.42 (0.95, 2.12) | 0.089 |
| **Female** | 6,285 | 190 |  |  |
| Social determinants of health (≥7) | 4,059 | 100 | Reference | Reference |
| Social determinants of health (<7) | 2,226 | 90 | 1.37 (1.01, 1.85) | 0.040 |
| **≥60 year** | 5,238 | 154 |  |  |
| Social determinants of health (≥7) | 3,234 | 70 | Reference | Reference |
| Social determinants of health (<7) | 2,004 | 84 | 1.62 (1.16, 2.26) | 0.004 |
| **<60 year** | 6,690 | 147 |  |  |
| Social determinants of health (≥7) | 5,072 | 101 | Reference | Reference |
| Social determinants of health (<7) | 1,618 | 46 | 1.20 (0.84, 1.72) | 0.326 |
| **Never drink** | 8,412 | 221 |  |  |
| Social determinants of health (≥7) | 5,697 | 119 | Reference | Reference |
| Social determinants of health (<7) | 2,715 | 102 | 1.51 (1.14, 1.99) | 0.004 |
| **Currently drinking** | 2,945 | 66 |  |  |
| Social determinants of health (≥7) | 2,243 | 45 | Reference | Reference |
| Social determinants of health (<7) | 702 | 21 | 1.07 (0.62, 1.87) | 0.803 |
| **Ever drunk** | 571 | 14 |  |  |
| Social determinants of health (≥7) | 366 | 7 | Reference | Reference |
| Social determinants of health (<7) | 205 | 7 | 1.41 (0.46, 4.27) | 0.546 |
| **Never smoked** | 7,308 | 202 |  |  |
| Social determinants of health (≥7) | 4,934 | 115 | Reference | Reference |
| Social determinants of health (<7) | 2,374 | 87 | 1.30 (0.97, 1.74) | 0.079 |
| **Currently smoking** | 3,594 | 82 |  |  |
| Social determinants of health(≥7) | 2,641 | 46 | Reference | Reference |
| Social determinants of health(<7) | 953 | 36 | 1.72 (1.09, 2.72) | 0.020 |
| **Ever smoked** | 1,026 | 17 |  |  |
| Social determinants of health(≥7) | 731 | 10 | Reference | Reference |
| Social determinants of health(<7) | 295 | 7 | 1.53 (0.54, 4.36) | 0.423 |
| **BMI<28** | 10,545 | 271 |  |  |
| Social determinants of health(≥7) | 7,258 | 154 | Reference | Reference |
| Social determinants of health(<7) | 3,287 | 117 | 1.34 (1.04, 1.73) | 0.024 |
| **BMI≥28** | 1,383 | 30 |  |  |
| Social determinants of health(≥7) | 1,048 | 17 | Reference | Reference |
| Social determinants of health(<7) | 335 | 13 | 2.13 (1.01, 4.48) | 0.047 |
| **Arthritis or rheumatism(Yes)** | 3,836 | 139 |  |  |
| Social determinants of health(≥7) | 2,289 | 72 | Reference | Reference |
| Social determinants of health(<7) | 1,547 | 67 | 1.21 (0.86, 1.71) | 0.271 |
| **Arthritis or rheumatism(No)** | 8,092 | 162 |  |  |
| Social determinants of health(≥7) | 6,017 | 99 | Reference | Reference |
| Social determinants of health(<7) | 2,075 | 63 | 1.63(1.17, 2.27) | 0.004 |

**Model1:** unadjusted; **Model 2:** adjusted for age, gender**; Model 3:** Model 2+adjusted for smoking status, drinking status, BMI, arthritis or rheumatism, hypertension, hyperlipidemia, diabetes mellitus, heart disease, CircS

**Abbreviation:** HR, hazard ratio; CI, confidence interval; CircS, circadian rhythm syndrome

**Supplementary Table 6 The association between social determinants of health and hip fracture with missing data.**

| **Characteristic** | **N** | **Event N** | **Model 1** | | **Model 2** | | **Model 3** | |
| --- | --- | --- | --- | --- | --- | --- | --- | --- |
|  |  |  | **HR (95%CI)** | **p-value** | **HR (95%CI)** | **p-value** | **HR (95%CI)** | **p-value** |
| Social determinants of health (Continuous) | 11,928 | 301 | 0.78 (0.71, 0.86) | <0.001 | 0.82 (0.74, 0.91) | <0.001 | 0.86 (0.77, 0.97) | 0.010 |
| Social determinants of health (Category) | 11,928 | 301 |  |  |  |  |  |  |
| Social determinants of health(≥7) | 3,641 |  | Reference | Reference | Reference | Reference | Reference | Reference |
| Social determinants of health(<7) | 8,287 |  | 1.89 (1.42, 2.53) | <0.001 | 1.68 (1.25, 2.26) | <0.001 | 1.42 (1.04, 1.96) | 0.029 |

**Model1:** unadjusted; **Model 2:** adjusted for age, gender**; Model 3:** Model 2+adjusted for smoking status, drinking status, BMI, arthritis or rheumatism, hypertension, hyperlipidemia, diabetes mellitus, heart disease, CircS

**Abbreviation:** HR, hazard ratio; CI, confidence interval; CircS, circadian rhythm syndrome

**Supplementary Table 7 The association between social determinants of health and hip fracture excluding cases within the first two years**

| **Characteristic** | **N** | **Event N** | **Model 1** | | **Model 2** | | **Model 3** | |
| --- | --- | --- | --- | --- | --- | --- | --- | --- |
|  |  |  | **HR (95%CI)** | **p-value** | **HR (95%CI)** | **p-value** | **HR (95%CI)** | **p-value** |
| **Social determinants of health (Continuous)** | 11,883 | 256 | 0.77 (0.69, 0.85) | <0.001 | 0.81 (0.72, 0.90) | <0.001 | 0.85 (0.76, 0.95) | 0.004 |
| **Social determinants of health (Category)** | 11,883 | 256 |  |  |  |  |  |  |
| Social determinants of health(≥7) | 8,278 |  | Reference | Reference | Reference | Reference | Reference | Reference |
| Social determinants of health(<7) | 3,605 |  | 1.83 (1.43, 2.34) | <0.001 | 1.62 (1.25, 2.09) | <0.001 | 1.44 (1.11, 1.87) | 0.006 |

**Model1:** unadjusted; **Model 2:** adjusted for age, gender**; Model 3:** Model 2+adjusted for smoking status, drinking status, BMI, arthritis or rheumatism, hypertension, hyperlipidemia, diabetes mellitus, heart disease, CircS

**Abbreviation:** HR, hazard ratio; CI, confidence interval; CircS, circadian rhythm syndrome

**Supplementary Table 8** ROC Curves of social determinants of health for hip fracture risk under varying metabolic and circadian rhythm conditions

| **Characteristic** | **Cutoff** | **AUC (95%CI)** | **Sensitivity** | **Specificity** | **PPV** | **NPV** | **P-value** |
| --- | --- | --- | --- | --- | --- | --- | --- |
| Total | -0.239 | 0.650 (0.620, 0.681) | 0.698 (0.646, 0.750) | 0.522 (0.513, 0.531) | 0.036 (0.032, 0.041) | 0.985 (0.982, 0.988) | <0.001 |
| Circs | 0.147 | 0.723 (0.663, 0.783) | 0.804 (0.695, 0.913) | 0.573 (0.550, 0.596) | 0.051 (0.036, 0.066) | 0.990 (0.984, 0.996) | <0.001 |
| Have no Circs | 0.071 | 0.641 (0.606, 0.676) | 0.440 (0.378, 0.502) | 0.764 (0.756, 0.773) | 0.045 (0.037, 0.054) | 0.982 (0.979, 0.985) | <0.001 |
| Mets | -0.064 | 0.731 (0.671, 0.791) | 0.746 (0.639, 0.854) | 0.645 (0.627, 0.663) | 0.049 (0.035, 0.062) | 0.991 (0.986, 0.995) | <0.001 |
| Have no Mets | 0.18 | 0.639 (0.604, 0.675) | 0.424 (0.362, 0.487) | 0.792 (0.783, 0.800) | 0.051 (0.041, 0.061) | 0.981 (0.978, 0.984) | <0.001 |
| Total | -0.239 | 0.650 (0.620, 0.681) | 0.698 (0.646, 0.750) | 0.522 (0.513, 0.531) | 0.036 (0.032, 0.041) | 0.985 (0.982, 0.988) | <0.001 |

**Abbreviation:** ROC, receiver operating characteristic curve; AUC, area under the curve; CI, confidence interval; MetS, metabolic syndrome; CircS, circadian syndrome; PPV, positive predictive value; NPV, negative predictive value
