# Supplementary material for: Exploring social determinants of health in the context of metabolic and circadian influences on new hip fracture risk: longitudinal insights from CHARLS
Source: BMC Public Health. 2025 Nov 21;25:4074. doi: 10.1186/s12889-025-25126-5 (PMC12639743; doi:10.1186/s12889-025-25126-5)
Supplement: Supplementary file 2 — Supplementary Material 2. [file 12889_2025_25126_MOESM2_ESM.docx]

**Trends in per capita disposable income by quintile among national residents (RMB Yuan per person), 2013-2023**

| **Quintile Group** | **2013** | **2014** | **2015** | **2016** | **2017** | **2018** | **2019** | **2020** | **2021** | **2022** | **2023** |
| --- | --- | --- | --- | --- | --- | --- | --- | --- | --- | --- | --- |
| **Low-income group** | ¥4,402 | ¥4,747 | ¥5,221 | ¥5,529 | ¥5,958 | ¥6,440 | ¥7,380 | ¥7,869 | ¥8,333 | ¥8,601 | ¥9,215 |
| **Lower-middle group** | ¥9,654 | ¥10,887 | ¥11,894 | ¥12,899 | ¥13,843 | ¥14,361 | ¥15,777 | ¥16,443 | ¥18,445 | ¥19,303 | ¥20,442 |
| **Middle-income group** | ¥15,698 | ¥17,631 | ¥19,320 | ¥20,924 | ¥22,495 | ¥23,189 | ¥25,035 | ¥26,249 | ¥29,053 | ¥30,598 | ¥32,195 |
| **Upper-middle group** | ¥24,361 | ¥26,937 | ¥29,438 | ¥31,990 | ¥34,547 | ¥36,471 | ¥39,230 | ¥41,172 | ¥44,949 | ¥47,397 | ¥50,220 |
| **High-income group** | ¥47,457 | ¥50,968 | ¥54,544 | ¥59,259 | ¥64,934 | ¥70,640 | ¥76,401 | ¥80,294 | ¥85,836 | ¥90,116 | ¥95,055 |

**Note:**

- Data were obtained from the integrated urban–rural household survey conducted by the National Bureau of Statistics of China (NBS) and compiled in the China Statistical Yearbook.
- At baseline, data were collected in 2011, when national per capita income levels were considerably lower than in subsequent years. Although quintile-specific national income data were not yet available under a unified urban–rural framework at that time, official statistics show that in 2011 the mean per capita disposable income was ¥21,810 in urban areas and ¥6,977 in rural areas. These figures indicate that a substantial portion of the population earned well below ¥9,000, making it a conservative yet contextually appropriate proxy for identifying lower-middle income status under the economic conditions of that year.
- Importantly, the China Health and Retirement Longitudinal Study (CHARLS) also adopted 2011 as its baseline survey year, with the first follow-up wave conducted in 2013. Coinciding with this, the National Bureau of Statistics (NBS) introduced a unified urban–rural household survey in 2013, which provided national quintile-level income data for the first time. According to these data, the mean per capita income of the second quintile (i.e., the lower-middle income group) in 2013 was ¥9,654. Given that national income levels in 2011 were significantly lower than in 2013, the ¥9,000 threshold used in our study lies just below this benchmark and thus serves as a methodologically sound and temporally coherent approximation for lower-middle income status at baseline.
- Each year, all households are ranked by per capita disposable income and divided into five equal quintiles (each representing 20% of the population). The table reports the mean per capita disposable income (in RMB) for the low-income, lower-middle, middle, upper-middle, and high-income groups.
- In 2011-2012, quintile-specific income statistics were not reported under a unified urban–rural framework. In 2012, only urban quintile data were released, whereas in 2011, only the mean per capita income for urban and rural residents were available. To ensure comparability, the main tables present national residents’ quintile series starting from 2013, when the National Bureau of Statistics launched the integrated urban–rural household survey, establishing a continuous and comparable quintile dataset.
- To further substantiate the selection of ¥9,000 as the per capita annual income threshold for identifying economically disadvantaged individuals, we emphasize its consistency with both historical baseline conditions and long-term stability in China's income distribution. At baseline (2011), national per capita income levels were markedly lower than in 2013, when the second quintile (the lower middle income group) had already reached ¥9,654. Accordingly, the ¥9,000 threshold serves as a conservative yet contextually appropriate proxy for lower middle income status under the prevailing economic conditions of 2011.
- As our study commenced in 2024, the most recent available data were from 2023. These data indicate that the low-income group, representing the bottom 20% of the population, had a mean per capita disposable income of ¥9,542, which is only slightly above the ¥9,000 threshold used in our analysis. This observation suggests that despite a decade of economic growth, the ¥9,000 cut-off remains a valid and empirically grounded proxy for identifying low-income status, thereby supporting the methodological soundness and practical relevance of our income classification.
